# Sex-Related Differences in the Diagnosis and Evolution of Parietal Cell Antibody-Positive Autoimmune Gastritis: A Large Single-Center Retrospective Cohort Study

**DOI:** 10.3390/diagnostics16030387

**Published:** 2026-01-26

**Authors:** Esteban Fuentes-Valenzuela, Santiago Blanco, Sergio Escribano Cruz, Javier Parra Villanueva, Almudena Calvache Rodríguez, Ariadna Gil Diaz, María del Carmen López-Martín, Itziar Rubio de la Plaza, Irene Chivato Martín-Falquina, Beatriz Rodríguez-Batllori Aran, Raquel Latorre Martínez, Luis Alonso Castillo Herrera, Daniel Alcalde Rodríguez, Karina Guzmán López, Alicia Bejerano Domínguez

**Affiliations:** 1 Gastroenterology Department, Hospital Universitario Infanta Elena, 28342 Valdemoro, Spain; 2 Health Research Institute, Fundación Jiménez Díaz University Hospital, Universidad Autónoma de Madrid (IIS-FJD, UAM), 28049 Madrid, Spain; 3 Autoimmune Laboratory, Hospital Universitario Fundación Jiménez Díaz, 28040 Madrid, Spain

**Keywords:** autoimmune gastritis, sex differences, autoimmune disorders

## Abstract

**Background/Objectives**: Autoimmune gastritis (AIG) is more common in females, although studies assessing the differences between females and males are scarce. Therefore, this retrospective observational study aimed to assess differences at diagnosis and the evolution of the full spectrum of AIG between females and males. **Methods**: A large retrospective single-center cohort study was performed. Two cohorts were included: patients newly diagnosed with AIG with positive parietal cell antibodies between June 2013 and June 2023, and those who underwent at least one follow-up endoscopy, constituted the second cohort. Both cohorts were categorized by sex. **Results**: A total of four hundred twenty-six patients were included. Three hundred seventeen were females (74.4%) and 109 males, with a median age of 53.5 years (IQR 44.7–65.3). Females were more likely to be non-smokers (71.5%, 226 patients versus 57.8%, 63 patients; *p* = 0.01), higher prevalence of autoimmune hypothyroidism (72 females, 22.7% versus nine males, 8.3%; *p* = 0.001), dyspepsia (130 individuals, 41% versus 28 individuals, 25.7%; *p* = 0.004), and iron deficiency anemia (100 females, 31.6% versus 18 males, 16.5%; *p* = 0.002), while vitamin B12 deficiency was higher amongst males (22, 21.6% versus 36; 11.6%; *p* = 0.012). Histological and endoscopic stages were similar at the diagnosis. The logistic regression identified iron deficiency anemia (OR 2.9; 95% CI 1.47–5.72; *p* = 0.002), dyspepsia (OR 2.3; 95% CI 1.28–3.9; *p* = 0.005), lower ferritin levels (OR 0.99; 95% CI 0.98–0.99; *p* < 0.001), and autoimmune hypothyroidism (OR 2.7; 95% CI 1.24–5.8, *p* = 0.012) associated with females at diagnosis. No significant differences were observed regarding progression to a worse stage (47 females, 39.1% versus 9 males, 28.1%; *p* = 0.26). **Conclusions**: In conclusion, this large retrospective study showed some differences in clinical factors associated with AIG, although the evolution was similar. Clinicians should be particularly vigilant for the diagnosis of AIG in female patients with iron deficiency anemia, dyspeptic symptoms, and autoimmune hypothyroidism.

## 1. Introduction

Autoimmune gastritis (AIG) is a persistent inflammatory condition that primarily targets the oxyntic mucosa, leading to atrophy of the gastric corpus. Its diagnosis is based on positive anti-parietal cell, anti-intrinsic antibodies, and histological examination, particularly of corporal mucosa. It shows some specific differences from Helicobacter pylori infection, such as corporal restricted atrophy, common pseudopyloric metaplasia, endocrine cell hyperplasia, and higher incidence of type I gastric neuroendocrine tumors for AIG [1].

The prevalence of the condition can fluctuate based on the diagnostic criteria applied, with an overall estimate reaching as high as 3.9%. When employing a combined approach of serological and histological diagnostics, the prevalence is reduced to approximately 1.8% [2].

Currently, there is a growing interest in elucidating etiopathogenesis, as well as the clinical and endoscopic diagnosis, and potential progression of this condition [3]. It might lead to a range of serious conditions, including malabsorption, vitamin deficiencies, and an increased risk of gastric malignancies, such as carcinoids or gastric adenocarcinoma.

Increasing evidence indicates that the disease may be progressive even in patients who initially lack significant atrophy on histopathological examination [4,5,6]. Recent literature has raised concerns regarding the incidence of gastric adenocarcinoma among patients diagnosed with autoimmune gastritis (AIG), indicating that this neoplastic risk may be attenuated in individuals who are negative for Helicobacter pylori infection, while the risk for type I neuroendocrine tumors is clearly increased [7,8]. For this reason, the current ESGE guidelines for the management of epithelial precancerous lesions and early neoplasia of the stomach (MAPS III) advocate for high-quality endoscopic surveillance every three years [9].

AIG exhibits specific epidemiological characteristics. It is clearly more common in females, with a female:male ratio of 2–3:1, in individuals older than 60 years and in patients with autoimmune disorders [10,11]. Sex-based differences in the diagnosis and progression have been well-documented in various autoimmune disorders, including inflammatory bowel disease [12,13]. These differences may rely on immunological differences as a more robust innate response, higher CD3+ and CD4+ T cells, increased response to viruses and vaccines, as well as cytokine production response to infections and humoral responses [14]. Thus, so far, only one study has assessed the differences, suggesting some specific clinical factors associated with females and males [10].

Therefore, this large, retrospective observational study aimed to assess differences at diagnosis and the evolution of PCA-positive AIG spectrum, including potential AIG, between females and males.

## 2. Materials and Methods

### 2.1. Study Design

A large retrospective single-center cohort study was performed at Hospital Universitario Infanta Elena, Valdemoro, Spain. This study included two cohorts: patients newly diagnosed with AIG between June 2013 and June 2023, and those who underwent at least one follow-up endoscopy constituted the second cohort. Baseline and follow-up data have been previously published [3], although for this analysis, both cohorts were categorized by sex. The study received approval from the Institutional Review Board of the Instituto de Investigación Fundación Jiménez Díaz (EO152-24_HIE) on 22 January 2025. Patient consent was waived due to the large retrospective review of the study, which was conducted according to clinical practice and did not involve any intervention in patients.

### 2.2. Participants

Inclusion criteria included: (1) Adult patients over 18 with (2) positive parietal cell antibodies (PCA) (≥1/160) (3) who had a gastroscopy with biopsies per Sydney protocol within ±1 year. Exclusion criteria were the following: (1) Helicobacter infection (HP) in biopsies, (2) positive fecal Helicobacter antigens (<12 months), (3) gastroscopy without biopsies, (4) or incomplete biopsies. Other (5) endoscopic or histological signs of drug-related causes or infections also led to exclusion. All patients who tested positive for HP within one year of the baseline endoscopy, whether through a positive stool test or histological assessment, were excluded from the study, regardless of whether they received treatment. Patients with prior HP (more than 12 months ago) and proper eradication were allowed in our study.

The difficulties associated with identifying seronegative AIG, along with the risk of selection bias inherent in including only those with more severe stages, led us to focus exclusively on patients with positive PCA.

The progression of autoimmune gastritis (AIG) was recorded whenever a patient underwent at least one follow-up gastroscopy, regardless of the indication for the procedure. Progression was defined as any transition to a higher histological stage at follow-up endoscopy, regardless of the magnitude of change. This binary definition (progression vs. no progression) was adopted to reflect the continuous nature of autoimmune gastritis evolution and to ensure sufficient statistical power for comparative analyses. A minimum interval of one year was required between the index endoscopy and any subsequent follow-up procedures.

### 2.3. Histological Assessment

Index endoscopy is defined as the first endoscopy performed within 1 year of a positive PCA. The decision to establish a ±1-year window between PCA testing and the index endoscopy was made to ensure a homogenous cohort concerning the timing of PCA evaluation.

Subsequent gastroscopies with biopsies, conducted in accordance with the Sydney protocol, were classified as follow-up procedures, necessitating a minimum interval of one year between them. However, because of the study’s retrospective design, the authors were unable to guarantee that all follow-up endoscopic evaluations adhered strictly to the established protocol guidelines.

Histological classifications were categorized into four stages according to characteristics observed exclusively in the oxyntic mucosa. The histological damage in our study encompassed chronic lymphocytic infiltrate, plasma cell infiltration, and atrophy of the oxyntic mucosa. This classification was based on a simplification of a previously published classification that assessed the evolution of AIG, allowing the inclusion of patients across the entire spectrum of histological stages [4]. Although it has not been formally validated, it offers a pragmatic operational framework for retrospective staging (and follow-up analyses) to make the retrospective intent unambiguous.

- Stage 0 (Potential): Positive PCA with no signs of chronic gastritis or atrophy.
- Stage 1 (Non-atrophic Gastritis): Positive PCA with lymphocytic and plasma cell infiltration.
- Stage 2 (Atrophic Gastritis): Positive PCA with lymphocytic and plasma cell infiltration and atrophy.
- Stage 3 (Complicated): Any prior stage with dysplasia, type 1 neuroendocrine tumors, intraepithelial glandular neoplasia, or adenocarcinoma. This stage should not be assessed solely based on the histological severity grade, nor does it supersede established atrophy-based classification systems.

All patients classified within stages 1, 2, and 3 were identified as “confirmed AIG” and stage 0 as “potential AIG”.

For histological analysis, stages were classified as advanced AIG when they corresponded to either stage II or stage III.

Additionally, pseudo-pyloric changes, intestinal metaplasia, and other polypoid lesions, including hyperplastic and fundic gland polyps, were recorded.

### 2.4. Data Measurement

The primary aim was to identify independent baseline factors associated with females. Secondary aims included evaluating differences in endoscopic and histological stages at diagnosis, as well as assessing the rate of progression to a more advanced histological stage at follow-up endoscopy. For the assessment of progression at the follow-up endoscopy, it was categorized as progression whenever a worse stage was noted, independently of the change. Anemia was considered whenever the level of hemoglobin was below 12.5 g/dL for females and 13 g/dL for males. Vitamin B12 deficiency was considered whenever the levels were below 160 pg/mL.

### 2.5. Statistical Methods

Continuous variables were presented as mean and standard deviation, and the median with interquartile range as warranted. The Shapiro–Wilk normality test was performed. Categorical variables were presented as numbers and percentages. Given the anticipated low sample size of advanced stage 3 disease cases, the specific histological characteristics were reported solely as absolute numbers without conducting any subsequent analyses.

A logistic regression was conducted to identify factors associated with female status at diagnosis. This analysis was conducted with an exploratory, association-focused objective and was not intended to develop or validate a predictive or diagnostic model. Model selection was based on the Akaike Information Criterion (AIC) to obtain the most parsimonious model. Results are presented as odds ratios (ORs) with 95% confidence intervals (CIs). Model performance indices, including sensitivity, specificity, positive and negative predictive values, and the area under the receiver operating characteristic curve (AUC), were calculated for descriptive purposes only and should be interpreted with caution, particularly in the context of the unequal sex distribution in the study population.

Kaplan–Meier analysis was performed to evaluate the evolution, using a Log-rank test to evaluate the differences between the two groups. For time-to-event analysis, the event was defined as the first documented progression to a worse stage. Time was calculated from the index endoscopy to the follow-up endoscopy, showing progression. Patients without progression were censored at the date of their last available endoscopy.

Statistical analyses were performed using Stata (StataCorp. 2016. Stata Statistical Software: Release 13. College Station, TX, USA: StataCorp LP). A *p* < 0.05 was considered statistically significant.

## 3. Results

During this period, a total of 1004 patients presented positive PCA, although 426 patients that fulfilled the study criteria and were included. Three hundred seventeen were females (74.4%) and 109 males, with a median age of 53.5 years (IQR 44.7–65.3). The median age at diagnosis was 52.8 (IQR 44.4–64.3) and 54.7 for males (IQR 47.1–67.7) (*p* = 0.24).

### 3.1. Differences in the Diagnosis

Baseline differences were identified between groups. Females were more likely to be non-smokers (71.5%, 226 patients) compared to males (57.8%, 63 patients; *p* = 0.01). Differences in autoimmune comorbidity were non-significant (121 females, 38.2% versus 31 males, 28.4%; *p* = 0.07), although females exhibited a higher prevalence of autoimmune hypothyroidism (72 females, 22.7% versus nine males, 8.3%; *p* = 0.001). Additionally, among symptoms associated with AIG diagnosis, dyspepsia was observed more frequently in female patients (130 individuals, 41%) than in males (28 individuals, 25.7%; *p* = 0.004). On the other hand, vitamin B12 deficiency was more common in males (50 males, 49% versus 103 females, 33.3%, *p* = 0.004). Notably, no differences in PCA concentration were observed. The remaining categorical baseline characteristics are shown in Table 1.

The median anti-intrinsic factor values were 2.7 (IQR 0.5–9.4) for females and 2.5 (IQR 0.5–5.45) for males (*p* = 0.9). At diagnosis, the median hemoglobin level was lower in females: 13.3 (IQR 12.1–14.1) for females and 14.9 (IQR 14–15.7) for males (*p* < 0.001). Median ferritin levels were 26 ng/mL (IQR 13–60) in females and 78 ng/mL (IQR 34–245) in males (*p* < 0.001). Iron deficiency anemia was more prevalent among females, affecting 31.6% (100 patients), compared with 16.5% (18 patients) among males (*p* = 0.002). Baseline vitamin B12 concentrations were higher among females: 292 pg/mL (IQR 204–395) for females and 266 pg/mL (IQR 162–392) for males (*p* = 0.046). Vitamin B9 values were 8.45 ng/mL (IQR 5.6–11.6) for females and 7.75 ng/mL (IQR 5.7–11.1) for males (*p* = 0.74).

### 3.2. Histological Stages at Diagnosis

At diagnosis, females and males exhibited similar histological stages, with a similar distribution of the different stages. Indeed, both sexes presented a similar rate of advanced AIG (158 females, 49.8% versus 57 males, 52.3%; *p* = 0.69). Moreover, the rate of corporal metaplasia was similar (140 females, 44.4% versus 56 males, 51.4%; *p* = 0.19), with a similar distribution of the type of metaplasia. The rest of specific distribution can be observed in Table 2.

### 3.3. Endoscopic Features at Baseline

The most common polyp detected at the diagnosis was hyperplastic polyps for both sexes (29 females, 9.2% versus 10, 9.2%; *p* = 0.8). The second most frequent polyps were fundic gland polyps, detected in seven females (2.2%) and one male (0.9%). No specific differences were observed between the groups regarding polypoid lesions.

While assessing the atrophy during the endoscopic, the most common presentation was corporal-restricted atrophy in both sexes (46 females, 15% versus 23 males, 22.6%; *p* = 0.33), with a similar presentation of the different extensions of atrophy (Table 2).

### 3.4. Factors Associated with Females at Diagnosis

The logistic regression identified that iron deficiency anemia (OR 2.9; 95% CI 1.47–5.72; *p* = 0.002), dyspepsia-associated symptoms (OR 2.3; 95% CI 1.28–3.9; *p* = 0.005), autoimmune hypothyroidism (OR 2.7; 95% CI 1.24–5.8, *p* = 0.012), lower ferritin levels (OR 0.99; 95% CI 0.98–0.99; *p* < 0.001) and lower odds of vitamin B12 deficiency (OR 0.47, 95% CI 0.27–0.8, *p* = 0.005) were associated with females at diagnosis (Table 3 and Table 4). This model presented a sensitivity of 97.4% and specificity of 26.5%, with a positive predictive value of 80%, negative predictive value of 77.1%, and an AUC of 0.78.

### 3.5. Follow-Up Endoscopy

A total of 153 patients underwent at least one follow-up endoscopy and were included in the follow-up cohort. Of these, 121 were female (78.9%) and 32 were male.

After a median follow-up period of 35.2 months (IQR 25.5–47.2), no significant differences were observed regarding progression to a worse stage. Specifically, 47 females (39.1%) and nine males (28.1%) experienced disease progression (*p* = 0.26). Cumulative risk of progression to a worse stage within 2, 3 years was 28.6% (95% CI 20.5–39), 67.2% (95% CI 54.9–78.9%) for females and 26.4% (95% CI 13.6–47.6), 40.4% (95% CI 22.3–65.4%) for males, respectively. 

**Figure 1:**
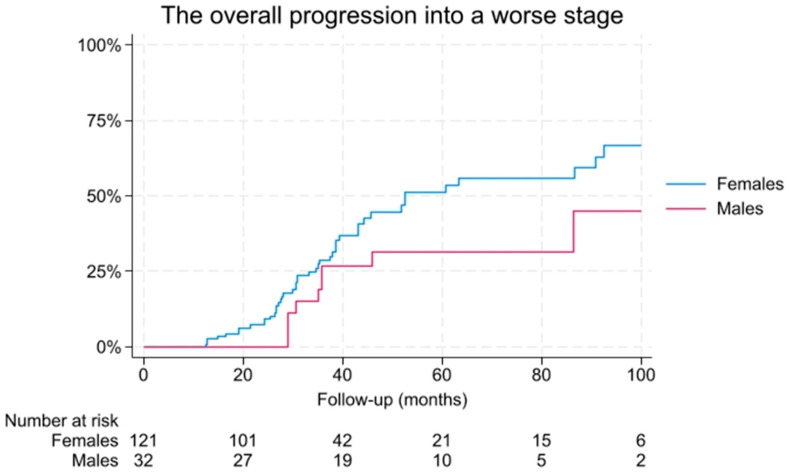
Kaplan–Meier assessment of evolution to a worse histological stage during the follow-up.

No significant differences were observed in the Kaplan–Meier analysis with the log-rank test (*p* = 0.08) (Figure 1). The absolute numbers of complicated diseases are shown in Table 5.

To evaluate the risk of progression to metaplastic changes, we observed that the rate of metaplasia was 44.4% at baseline, which increased to 53.6% at the follow-up assessment (*p* = 0.06). During this period, a progression to metaplastic changes was noted in 12.4% of females (95% CI: 0.1–24.9) and 3.1% of males, with a significant difference of 9.2% (95% CI: 6.1–12.4; *p* = 0.001) between females and males from baseline to follow-up endoscopy.

The rate of advanced AIG from baseline to the follow-up endoscopy increased to a total of 21% for females (95% CI 8.6–32.9) and 12.5% for males (95% CI −11.8–36.8), with a statistical difference of 8.2% (95% CI 5.1–11.2; *p* = 0.001) between both.

## 4. Discussion

In this retrospective cohort analysis evaluating sex-based disparities in the diagnosis and clinical course of the entire PCA-positive AIG spectrum, several clinical variables were identified as being predominantly associated with female patients at diagnosis. These included a higher prevalence of iron deficiency anemia, dyspeptic symptoms, coexisting autoimmune hypothyroidism, and decreased serum ferritin concentrations. Importantly, there were no statistically significant differences between female and male subjects regarding endoscopic or histopathological features either at initial evaluation or throughout follow-up.

Autoimmune diseases may affect up to 8% of the population, with approximately 78% of affected individuals being women. The sex distribution varies among specific conditions; for example, Sjögren’s syndrome demonstrates a pronounced female predominance (19:1 ratio), whereas type 1 diabetes shows a more balanced distribution, and ulcerative colitis or Crohn’s disease are more common in men. These differences are thought to be influenced by the higher frequency of humoral and autoantibody-driven responses in females, while T cell-driven responses show less sex disparity [15]. In other autoimmune clinical outcomes, differences are exhibited, such as for psoriasis, which presents lower PASI scores, with men more often requiring biologic therapy [16]. Similarly, the ENEIDA registry study showed that females demonstrated a higher median disease onset and later disease onset, extensive colonic involvement, and extraintestinal manifestations [13].

Although many studies about AIG have been published in recent years in different populations, including large prospective and retrospective studies, the differences regarding the diagnosis and evolution stratified by sex are not included [1,5,7,17,18,19,20].

A retrospective cross-sectional study of 435 subjects by Lahner et al. identified specific sex-differences, although evolution was not assessed. Multivariate logistic regression demonstrated that female patients demonstrated significantly higher prevalence of iron deficiency anemia (odds ratio [OR] 3.6), body mass index (BMI) < 25 kg/m^2^ (OR 3.1), coexisting autoimmune thyroid disease (OR 2.5), and dyspepsia (OR 2.4). In contrast, male patients showed significant prevalence of tobacco use (OR 1.8) and pernicious anemia (OR 1.7) [10]. In another retrospective conducted by Wang et al. that included 183 patients, found that females presented a higher prevalence of enterochromaffin-like cell hyperplasia (69.4% vs. 39.2%) and iron deficiency anemia (25.0% vs. 0.0%), and were more likely to have autoimmune hypothyroidism [21]. Similarly, our study confirmed that iron deficiency anemia (OR 2.9), dyspepsia-associated symptoms (OR 2.3), lower ferritin levels (OR 0.99), increased autoimmune hypothyroidism (OR 2.7), and lower odds of vitamin B12 (OR 0.47) were associated with females at diagnosis with an acceptable AUC. However, our logistic regression analysis was designed to explore associations between baseline clinical characteristics and female sex, rather than to develop a predictive or diagnostic model. Consequently, the reported odds ratios should be interpreted as measures of association within this cohort. Moreover, although the model demonstrated a high sensitivity (97.4%), this was accompanied by a low specificity (26.5%). Moreover, the higher prevalence of iron deficiency anemia and lower ferritin levels in female patients should be interpreted cautiously. These findings may reflect physiological differences, especially premenopausally. While the age at diagnosis is similar for both sexes, data on menopausal status were not consistently available, limiting further analysis. Thus, while iron deficiency is an important indicator for diagnosing autoimmune gastritis in women, these differences do not imply sex-specific disease mechanisms and should be interpreted cautiously. Conversely, it is important to note that our cohort was exclusively composed of patients who tested positive for parietal cell antibodies, and we specifically excluded individuals with active Helicobacter pylori infections. Additionally, we did not evaluate other genetic or immunological differences that could play a role in our findings.

Regarding the evolution, a recent prospective study conducted by Miceli et al. that included 498 patients with AIG found that males were associated with a higher likelihood of evolving from potential to overt AIG, including all the different histological stages with confirmed histopathological features of AIG [4]. In contrast, our study showed no differences in the evolution when females and males were compared. This may be attributable to the reduced sample size of the follow-up cohort in our study. Moreover, the interpretation of these results requires caution, particularly regarding extrapolation, due to our reliance on a four-stage simplified histological classification derived from the five-stage system proposed by Miceli et al. This classification has yet to undergo validation in the context of AIG.

Nevertheless, there are several limitations that should be mentioned. Firstly, it is a retrospective study with its inherent risk of selection bias. Secondly, not all patients present a follow-up endoscopy, and therefore, the follow-up data should be interpreted with caution. Thirdly, only patients with a positive serology were included in this study, although up to 20% of patients with AIG could present a negative serology [17]. Fourthly, beyond gross endoscopic appearance, other endoscopic features were not evaluated due to the difficulties in obtaining other items in the endoscopic reports. Therefore, a more granular analysis of lesion topography and morphology was not feasible due to the retrospective design and the lack of standardized reporting of lesion location (corpus, antrum, or incisura) and appearance (polypoid vs. non-polypoid). Fifth, not all follow-up endoscopies might be protocol-drive and some of them might be due to symptoms, due to the retrospective design. Lastly, OLGA/OLGIM classifications were not evaluated in our study. This omission merits clarification, as our investigation was focused on AIG with a retrospective design. The essential comprehensive and standardized histological mapping of both the antral and corporal regions necessary for accurate OLGA/OLGIM scoring was not uniformly available in the histological reports we reviewed. This limitation compromised the feasibility and reliability of applying these scoring systems to our cohort.

On the other hand, the main strength of this study lies in its substantial sample size, establishing it as one of the most comprehensive investigations of sex-related factors at both diagnosis and during follow-up. Furthermore, the analysis elucidated specific features associated with female patients at the time of diagnosis. Nonetheless, the lack of a control group without AIG constrains the generalizability of these findings to the general population.

## 5. Conclusions

In conclusion, this large retrospective study showed some differences in clinical factors associated with AIG, although the evolution was similar. Clinicians should be particularly vigilant for the diagnosis of AIG in female patients with iron-deficiency anemia, dyspeptic symptoms, and autoimmune hypothyroidism.

## Figures and Tables

**Table 1:** Comparison of baseline characteristics at the diagnosis.

	Females N = 317	Males N = 109	*p* Value
**Smoking habit, n (%)** **Former smoker** **Smoker** **Non-smoker**	40 (12.7) 50 (15.8) 226 (71.5)	28 (25.7) 18 (16.5) 63 (57.8)	**0.01**
**Family history of gastric cancer, n (%)** **First degree** **Second degree**	6 (1.9) 6 (1.9)	4 (3.7) 1 (0.9)	0.5
**Autoimmune comorbidity, n (%)**	121 (38.2)	31 (28.4)	0.07
**Autoimmune hypothyroidism, n (%)**	72 (22.7)	9 (8.3)	**0.001**
**Previous Helicobacter pylori eradication, n (%)**	62 (19.6)	18 (16.5)	0.48
**Parietal cell autoantibodies, n (%)** **1/160** **1/320** **1/640** **1/1280**	69 (21.8) 114 (36) 82 (25.9) 52 (16.4)	31 (28.4) 37 (33.9) 29 (26.6) 12 (11)	0.36
**Associated symptoms, n (%)**	160 (50.5)	48 (44)	0.246
**Dyspepsia, n (%)**	130 (41)	28 (25.7)	**0.004**
**Iron deficiency anemia** **, n (%)**	100 (31.6)	18 (16.5)	**0.002**
**Megaloblastic anemia** **, n (%)**	11 (3.5)	8 (7.3)	0.08
**Vitamin B12 deficiency, n (%)**	103 (33.2)	50 (49)	**0.004**
**Iron deficiency** **, n (%)**	9 (2.8)	2 (1.8)	0.56

**Table 2:** Comparison of the baseline endoscopic and histological features of autoimmune gastritis at the time of diagnosis.

	Females	Males	
**Stage 0** **Stage 1** **Stage 2** **Stage 3**	80 (25.2) 75 (23.7) 157 (49.8) 5 (1.6)	25 (22.9) 24 (22) 57 (52.3) 3 (2.8)	0.69
**Potential AIG, n (%)**	80 (25.2)	25 (22.9)	0.63
**Confirmed AIG, n (%).**	237 (74.8)	84 (77.1)	0.63
**Advanced AIG** **, n (%)**	158 (49.5)	57 (52.3)	0.69
**Corporal metaplasia** **, n (%)**	140 (44.4)	56 (51.4)	0.19
**Type of metaplasia, n (%)** **Intestinal** **Pseudopyloric** **Intestinal + pseudopyloric**	98 (30.9) 22 (6.9) 20 (6.3)	41 (37.9) 7 (6.4) 8 (7.3)	0.56
**Polyps, n (%)** **Hyperplastic** **Fundic gland** **Adenoma** **Neuroendocrine tumor**	29 (9.2) 7 (2.2) 3 (1) 2 (0.6)	10 (9.2) 1 (0.9) 0 0	0.8
**Atrophy, n (%)** **Corporal** **Fundus + corporal** **Antral, corporal + fundus** **Antral + corporal** **Antral**	46 (15) 46 (15) 27 (8.8) 24 (7.8) 12 (3.9)	23 (22.6) 11 (10.8) 9 (8.8) 9 (8.8) 1 (1)	0.33

**Table 3:** Specific histological features of complicated disease. The absolute numbers are shown.

	Females; n = 5	Males; n = 3
**Dysplasia**	2	3
**Adenocarcinoma**	1	0
**Neuroendocrine tumor.**	2	0

**Table 4:** Logistic regression to identify factors associated with females at the time of diagnosis.

	Odds Ratio	95% Confidence Interval	*p* Value
**Iron deficiency anaemia**	2.9	1.47–5.72	0.002
**Dyspepsia**	2.3	1.28–3.9	0.005
**Ferritin**	0.99	0.98–0.99	<0.001
**Vitamin B12 deficiency**	0.47	0.27–0.8	0.005
**Autoimmune hypothyroidism**	2.7	1.24–5.8	0.012

**Table 5:** Specific histological features of complicated disease at the follow-up endoscopy. The absolute numbers are shown.

	Females	Males
**Dysplasia**	2	0
**Adenocarcinoma**	1	0
**Neuroendocrine tumor.**	2	0

## Data Availability

The original contributions presented in this study are included in the article. Further inquiries can be directed to the corresponding author.

## Abbreviations

The following abbreviations are used in this manuscript:

AIG	Autoimmune gastritis
PCA	Parietal cell antibodies
IQR	Interquartile range
HP	Helicobacter pylori
